# Process, Practice and Progress: A Case Study of the Health Impact Assessment (HIA) of Brexit in Wales

**DOI:** 10.3390/ijerph17186652

**Published:** 2020-09-12

**Authors:** Liz Green, Kathryn Ashton, Nerys Edmonds, Sumina Azam

**Affiliations:** 1 Policy and International Health, WHO Collaborating Centre on Investment for Health & Wellbeing, Public Health Wales, Cardiff CF10 4BZ, UK; kathryn.ashton2@wales.nhs.uk (K.A.); nerys.s.edmonds@wales.nhs.uk (N.E.); sumina.azam@wales.nhs.uk (S.A.); 2 Faculty of Health, Medicine & Life Sciences, Maastricht University, Duboisdomein 30, 6229 GT Maastricht, The Netherlands

**Keywords:** health impact assessment, Brexit, health and well-being

## Abstract

Health impact assessment (HIA) is a systematic and flexible tool, which is advocated by the World Health Organisation as a method through which to consider the impact of policies on the health and well-being of a population, and the inequalities that may arise because of it. In 2018, the HIA support unit in Wales carried out a comprehensive and unique HIA on the impact of Brexit in Wales. The aims were to understand the differential impacts that Brexit would have on the health and well-being of the population and to provide evidence to inform decision makers across a range of public bodies. It followed a five-step process for HIA and utilised a wide range of evidence sources and health intelligence including both quantitative and qualitative evidence. This paper reflects on the process of carrying out the HIA and the methods used. It discusses the stages of the HIA, and shares the findings and reflections of implementation which will be beneficial to other HIA practitioners and policy makers. It does not concentrate on the findings of the HIA in detail, but focusses on what worked and any challenges encountered. It has been used to progress the practice of HIA in Wales and demonstrates the value of HIA as a method to inform and influence complex decisions.

## 1. Introduction

Health and well-being is majorly influenced by factors outside of health systems, such as spatial planning, the economy and governmental policies and their decisions, plans and policies [1]. These factors are routinely referred to as the “social determinants of health” and have been articulated in frameworks such as that depicted by Dahlgren and Whitehead [2]. The social determinants are multifaceted and need to be addressed in order to tackle the “causes of the causes” of ill health [3,4] which are often policy driven, as the impact of them can create or exacerbate existing inequalities in social and health outcomes for a wide range of population groups [5]. One of the key concepts advocated to address this is to consider the health implications of policies in a range of settings, sectors and systems in order to protect and promote health and improve health outcomes. This is known as health in all policies (HiAP) [6,7,8]. HiAP entails working synergistically across sectors to take account of the health implications of decisions, aims to avoid health harm and rebalance social and health equity [8]. As part of this approach to protecting health and reducing inequalities, health impact assessment (HIA) is a tool commonly advocated to do this [9,10].

HIA is defined as “a combination of procedures, methods and tools by which a policy, plan or project may be judged as to its potential effects on the health of a population, and the distribution of those effects within a population” [11,12]. Underpinned by the World Health Organisation (WHO) holistic definition of health and well-being, it uses the social determinants of health as a lens through which to identify health impacts [13]. As a process, HIA is a systematic and scalable which allows health and well-being to be considered in all policy sectors in a flexible way. The process explicitly raises awareness of well-being and inequalities [11] and is recommended by the WHO as an important method with which to drive and apply HiAP to support healthy public policy-making [8]. HIA is explicitly tied to HiAP as it can enable collaboration across sectors to influence and inform decision-making processes [11], identifies positive and negative impacts on the population and makes recommendations to mitigate and avoid them [12]. However, previous evidence acknowledges there can be both benefits and challenges attached to the use of HIA dependent on context [14,15,16].

In 2018, the United Kingdom withdrew from the European Union (EU), routinely referred to as “Brexit” [17,18]. This was an unprecedented event in UK history, and as such the impact was not yet known for a wide range of policy areas, including the impact on the health system but also the economic and social impact. These impacts may not be distributed evenly across the population and therefore increase inequalities. To date, little has been published in the peer-reviewed literature to capture the knowledge and learning from carrying out an HIA on an event such as Brexit [19]. This paper aims to explore the use of HIA to examine the impact of Brexit in Wales on health, well-being and inequalities. Wales is a devolved nation, with its own government and Parliament, and as such has the ability to legislate in specific areas including for health, planning and the environment. It has an underpinning tenet of sustainable development, which is built on the platform of the Well-being of the Future Generations (Wales) Act 2015 (WFGA) [20]. This Act has a clear focus on addressing health, social and economic inequalities and provides an enabling environment to promote an HiAP approach to policy-making. The Act consists of seven core well-being goals including resilient communities, economy and culture, which Welsh public bodies must strive to maximise. In including goals for “A more equal Wales” and “A healthier Wales”, the Act enshrines a strategic commitment to considering and integrating health and well-being into policies across sectors and addressing inequalities. The Act also specifies that this must be carried out using five ways of working, which are prevention, involvement, long-term thinking, integration and collaboration.

This paper outlines the case study of a complex, comprehensive HIA carried out in Wales, describes the process followed, the impact of the report and discusses and reflects on the knowledge obtained from undertaking the process by demonstrating the implementation of HiAP via HIA in our context. As such, the results of the HIA will not be detailed in any depth. This paper examines the benefits and issues that can arise from carrying out an HIA on a novel subject such as Brexit and what can be learnt in order to evolve practice and mobilise HiAP at a regional level in a nation like Wales. This paper is relevant to policy makers and practitioners who may be required to carry out HIAs on significant policies, major challenging and unique events and it aims to share the transferable learning. It can also demonstrate the “added value” of carrying out a process such as HIA on policies and exceptional events such as Brexit.

## 2. Materials and Methods

HIA in Wales is based on the WHO definition of health and is carried out as a flexible and systematic evidence-based process [13,21]. Supported by the Wales Health Impact Assessment Support Unit (WHIASU) and Public Health Wales (PHW), the process uses the determinants of health as a lens through which to assess the impact of policies, plans or projects. It follows a broad mixed methods approach to HIA, considers the potential positive and the negative (and unintended negative) impacts across the breadth of determinants, and focuses on the impact on health equity and vulnerable populations. It does this via two checklists that focus on population groups and the wider determinants of health and well-being [21]. The HIA process also directly involves organisations, public bodies and communities (and their representatives) who could, or will be, affected by the policy or plan under assessment or have an interest in it.

The Brexit HIA was undertaken over a six-month period in 2018. It captured the impacts of Brexit at that moment in time for Wales and was set against an evolving political situation predating the date of withdrawal. Evidence in relation to Brexit and its differential scenarios was limited as the HIA was carried out, but modelling scenarios and other impact assessments have since been published into 2019 and early 2020 [22,23,24]. Based on the context at the time, the HIA did not appraise specific Brexit scenarios (for example, a withdrawal with major alignment with many EU regulations and trading positions versus a “no-deal” agreement) because of the short timeframe and complicated nature of Brexit. It therefore explicitly focussed on the impacts that could potentially occur in Wales and those groups that could be affected by the UK exiting from the current EU framework.

The HIA followed the Welsh HIA guidance [21], which is based on standard international practice with a five-stage process and was comprehensive, participatory and prospective in its nature.

### 2.1. Screening and Scoping

A core group and a working group were established to carry out the HIA. A strategic advisory group (SAG) was formed to provide governance to the HIA. The SAG was cross-sector and disciplinary in nature and consisted of highly relevant internal and external stakeholders, for example from PHW, Welsh Government (WG), academia and from a range of backgrounds (environmental public health; health policy, Brexit planning). In addition to providing oversight of the process, the SAG also provided guidance and advice, agreed on the findings and reviewed and made suggestions for the developing HIA report.

A screening paper was developed by the working group that highlighted the potential determinants of health that could be affected, the vulnerable groups and policy areas that could be affected by Brexit, which covered the whole population. In addition, a scoping paper was drafted. It defined the scope of the HIA based on the information from the screening paper and included the timescales, the types of evidence needed and the parameters of the process in order to ensure that the HIA was robust and high quality. This was carried out in tandem with screening to expedite the process, articulate those to be involved and the resources needed.

A prioritization process of the impacts was carried out due to the huge range, sensitivity, complexity, depth and breadth of potential impact of Brexit across all the determinants and populations in Wales, and its effect with respect to inequalities and the need to act quickly. A list of prioritization criteria was developed and included any evidence relevant to Wales of direct impact on health and well-being, and the strength of the potential impact. The SAG prioritised determinants such as healthcare, research and development, and health protection and food—safety, supply, access, and mental well-being. Populations such as young people, adults of working age and non-UK EU nationals were also prioritised due to the potential negative impact of Brexit on them. An example of the completed list of prioritised determinants is contained in Table A1.

### 2.2. Evidence

A literature review was undertaken using high quality databases (ProQuest, MEDLINE and Embase) to review the potential impact of Brexit on health and the economy. It focussed on peer-reviewed evidence alongside a review of grey literature, which focussed on impacts such as the healthcare, EU funding and the environment. Only studies included were those that focussed on the impact of Brexit across the population of Wales and the UK, published in the English language since January 2017, and had health determinant, well-being and equity outcomes. All results were screened and reviewed by two independent reviewers.

Quantitative health intelligence data were gathered and a community health profile was constructed based on the findings of the screening and scoping stages. It contained robust technical and quantitative data from 2017 and 2018 in relation to the demographics of Wales, health intelligence statistics in relation to the prevalence of health conditions in Wales (Welsh Health Survey [25] and levels of deprivation (Welsh Index of Multiple Deprivation) [26].

Semi structured qualitative interviews (n = 17) were completed involving 25 representatives from 12 organisations. Some organisations had more than one individual contribute to the interviews dependent on their field of expertise. Interviewees were selected via purposeful sampling and in total 20 organisations were approached. Interview respondents included representatives from a wide range of identified affected sectors, including the Welsh NHS Confederation, Nursing and General Practice, WG, Food Standards Agency Wales and the Local Government. The interviews aimed to identify organisations’ preparedness for the UK’s exit from the EU and identify which particular vulnerable groups they believed would potentially be affected alongside which social determinants could be impacted. Post an initial rapid literature review, a set of open-ended interview questions were developed to facilitate the discussion. They did not include obtaining the political views of the organisations or representatives about Brexit. The interviews were carried out in person with the exception of one, which was carried out via telephone.

In addition to the interviews, a cross-sector, multidisciplinary participatory stakeholder workshop was held on the 3 October 2018. A wide range of stakeholders were invited (n = 35) to contribute and in total 14 people attended. The purpose of the workshop was to capture any qualitative evidence and contextual knowledge from the organisations, sectors and community representatives about how Brexit could affect them. This included individuals from a range of disciplines and agencies including environmental and public health, sustainable development, healthcare services and the housing sectors. It was independently facilitated by the WHIASU leads and carried out in an interactive and transparent manner. Participants agreed to the findings being reported. A transcribed record of the workshop was completed and sent to the participants for agreement and consent. The workshop participants completed a feedback and evaluation form. No ethics approval was required for this work. The transcripts from the interviews and the workshop were integrated into the overall collated evidence to provide a rich, wide-ranging overview of the potential impact of Brexit for Wales in the near and long term.

### 2.3. Appraisal

During four half-day sessions, all evidence was collated, synthesised and analysed by individuals who led the HIA, gathered the health intelligence and carried out the interviews and literature review. As in usual HIA practice [21], a robust discussion took place to make sense of all the evidence gathered. Two matrices were created as part of the key papers at the start of the process and it was these that were completed as discussions took place and gave the analysis structure. The impact considered was for the identified population groups and the prioritised determinants of health. Evidence was identified and discussed to support the characterisation of the impact, for example, duration of impact. The matrix also listed the policy pathways through which the impact would manifest itself. These were drawn up after discussions about how Brexit would impact on the population and constructed on advice of a SAG member. The team realised that it was not actually Brexit that would have the impact but that any impact would occur thorough policy pathways; for example, the curtailment of the free movement of people from the EU could have major implications for staffing in health and social care services because high levels of non-UK nationals work in these sectors. The full characterisation was summarised as described in Table 1.

### 2.4. Reporting

The evidence, analysis and findings were written up with the SAG given opportunities to review and comment on the draft and final reports. Internally in PHW, it was reviewed by the Board and subject to quality assurance process using the WHIASU Quality Assurance review framework [27].

### 2.5. Monitoring and Evaluation including Review and Reflection

Monitoring and evaluation indicators were established and included, for example, tracking and collating newly published evidence in the short term and the impact on key determinants such as mental well-being in the long-term. This monitoring is still ongoing and updated approximately every six months. A review and reflection meeting was held post publication to capture any learning. It consisted of the working group and the findings have informed some of this paper.

## 3. Results

### 3.1. Findings of the HIA

The purpose of this paper is not to report the findings of the HIA itself, but to focus on the actual methods and process of the HIA in order to share learning and inform policy and decision makers and practitioners. To aid understanding, however, the main findings of the HIA are summarised.

The HIA identified major, direct potential impacts across the social determinants of health and well-being including for health and social care, and environmental regulations such as food safety and supply. Many of these are potentially probable, major and negative in their character. Uncertainty, the changing nature of the UK’s relationship with the EU, economic downturn and changes in regulatory alignment were identified as key pathways for health impacts. Wider socioeconomic impacts included the impact on working conditions and the loss of access to EU economic and social investment funding on deprived communities. Community impacts included the potential for divisions in communities from the “remain/leave” vote and the potential for increased hate crime against non-UK EU nationals. A major indirect determinant affected by Brexit was identified as mental well-being with outcomes being potentially both positive and negative. Some opportunities were identified based on devolution and the ability for Wales to develop sustainable policies, for example, land management, environment and health policy through the WFGA.

The HIA also highlighted inequalities and impacts across vulnerable population groups, such as Welsh geographical areas receiving EU funding: farmers and farming communities, young people, older people, working age population in areas that rely on one employer and whom may be affected by an economic downturn or trade tariffs leading to mass unemployment events [28].

Future free trade agreements and trade arrangements were noted as key determinants of health and well-being. This was because future trade agreements can have a major effect on the economy, can provide favourable or nonfavourable conditions for major employers in Wales, such as Airbus UK, which are also major local employers.

The assessment also identified gaps in the evidence base and recommended further research in those areas; this included the impact of Brexit on mental well-being (particularly in relation to some population groups such as young people or farmers and farming communities). A set of recommendations proposed for action were completed and included, for example, the need to utilise the HIA as a joint organisational framework to coordinate, develop and track actions, monitoring the identified impacts and that actions and policies should be prioritized so that the impacts on population groups are addressed in order to mitigate for potential increased inequalities.

### 3.2. Reporting, Review and Reflection (Including Monitoring and Evaluation)

The HIA was published in Welsh and English, and consisted of an Executive Summary providing an overview of the HIA and key findings, a Main Report providing an appraisal and analysis of the evidence, recommendations and future actions that could be implemented, and a Technical Report in two parts that provided a comprehensive overview of the HIA methodology, all the evidence, the tools and checklists used in the HIA and the matrices detailing the evidence of impact across the population groups and the determinants of health and well-being [29]. All the evidence was openly published and supports principles of HIA such as the ethical use of evidence and transparency [30].

A “review and reflection” meeting was held by the working group in March 2019 to discuss key questions such as how the HIA influenced decision makers and how its recommendations were actioned. The overall review, reflection and monitoring of the Brexit HIA will be a multistage process over time, taking into account short- to long-term impacts and any outcomes. To date, the HIA has had an impact in Wales with research funding coming onstream for PHW to carry out research on the impact of Brexit on farmers and young people [28], and to organize a symposium on “trade and health and well-being” that was held in November 2019 [31]. Many of the recommendations have been actioned in the 18 months post publication; for example, a high level cross-organisational strategic workshop was held by PHW and WG in August 2019 to discuss the immediate actions needed to influence future policies, strategies and plans to maximise the long-term positive opportunities for Wales that will emerge from Brexit. Many stakeholders took away actions to implement and conversations to be followed up by using the Brexit HIA as a lens. It has been very positively received politically. In the Senedd (Welsh Parliament) debate on Brexit in January 2019, Vaughan Gethin, the Welsh Health Minister, praised the HIA and its usefulness to WG [32]. This was due to the distinct lens it applies and because it provides robust evidence for national and local decision makers and planners to plan for and respond to Brexit at a time when little robust evidence-based information was available.

## 4. Discussion

The HIA outlined in this paper examined the potential impact of Brexit on the population of Wales and future health and well-being outcomes across key determinants. It identified the potential positive or negative impacts of Brexit in relation to these and highlighted the potential inequalities that could arise from Brexit across the population in Wales. It appraised a wide range of evidence and provided information and recommendations to support policy decisions and future actions to address the impact made by PHW and key strategic partners such as the WG. In addition, it demonstrated the implementation of HiAP in practice by considering harms to health, informing decisions and working collaboratively with key stakeholders and sectors.

The Brexit HIA has helped to clarify and progress both HIA and HiAP in Wales. The WG strongly supports HiAP using HIA as a method by which to improve health and health outcomes and address inequalities. In 2017, the WG passed a public health act [33] that legislates for HIA to be carried out by public bodies in specific circumstances. This HIA reinforced the importance of the HIA process to WG and has demonstrated the value and benefits of HIA to a wide range of senior leaders and planners in a broad range of public bodies in Wales, and has been informative at a time when there was little robust information forthcoming about the impact of Brexit in relation to wider public health and healthcare services.

The evidence produced by this HIA changed many people’s perspectives on HiAP and HIA and created more advocates for the process from one of theory to actual practice through its analysis of evidence and usefulness to them. The creation of strategic advocates or “champions” is stressed as an important and essential driver for health and equity [34,35,36]. It has promoted the continued support of HIA at a senior strategic level through advocacy and support for HIAs to seeing the actual benefits of the method in reality. This is clearly demonstrated in PHW with the commissioning of an HIA on climate change in Wales (forthcoming 2020) but also a series of HIAs in response to the COVID-19 response policies such as “lockdown” and any changes that may arise from it [37]. The concept of HIA as an important tool to inform and support decisions, guide the development of organisational planning for complex strategic priorities and to act on the information and recommendations; i.e., the HIA highlighted areas for future action, strengthening opportunities to be explored or research that has subsequently been carried out [28]. Its importance and value to decision makers has led to follow-up work, including the publication of a short rapid review and update report at the six-month point [38].

It has also promoted practice-based professional learning, which is essential to develop skills and knowledge, and is required as part of membership in professional bodies [39,40]. Key findings from the reflection meeting were that the experience of the leads (HIA experts) and team (policy researcher and public health practitioners) was invaluable. The team all knew their roles and communication was very good between them. For team members new to HIA, it provided them with a better understanding the method, its component parts, evidence and data needed, and how it can be helpful for organisations, for example, by including recommendations for mitigation to address the identified negative impacts. It also demonstrates how HIA can be utilised to implement HiAP in practice as it achieved its aim of identifying the potential impact of Brexit in Wales over the short to long term on key determinants and the populations affected, thereby informing integrated action through collaboration with stakeholders and mitigation for any negative impacts.

This HIA of Brexit in Wales was unique in approach, differing from standard HIA practice that tends to focus on one single policy area or proposal. HIA is frequently described as an iterative process [15,21] but is often depicted in figures and guidance with a clearly linear process flowchart to describe the order and steps to take [41,42,43]. However, by its very nature Brexit encompasses many policy areas, complex international relations and uncertainty. As a result, the Brexit HIA was scoped alongside screening due to the need to reflect on and guide discussions about the resources needed, and to ensure the report would provide timely information for decision makers. It was also used as a method to quickly identify and inform the leads about the stakeholders to involve in the process. The scope of this HIA was tight in approach due to factors such as the deadline for withdrawal and the potential breadth of the types of Brexit that could occur (for example, “soft” Brexit). This HIA tested the HIA methods and proven the flexible and adaptable nature of the process. The multidisciplinary, cross-sector SAG and working group were both valuable within the scoping stage of the HIA due to their expertise, their ability to prioritise impact and their input into the construction of policy pathways.

In HIAs, the last stage is commonly listed as monitoring and evaluation. However, in practice, it is rarely carried out [43,44,45] or if it is, it is contained within the health components of many Environmental Impact Assessments (EIA) and their health management and monitoring plans [45], which tend to monitor indicators for biophysical health or health services. In Wales, many participatory HIAs are routinely process evaluated [46,47,48], but this Brexit HIA gave the leads the opportunity to ensure that a strong review, including monitoring and evaluation indicators, of both the process and its impact could take place. The Brexit HIA identified a number of indicators to monitor, i.e., mental well-being and questions in the Welsh Health Survey [25] such as “how positive do you feel about the future?” in relation to supporting the monitoring of the HIA.

The terminology used to describe the final stage of an HIA, previously referred to in Wales as “monitoring and evaluation” [21], has evolved over recent years in Wales to become “review and reflection” (which includes monitoring and evaluation) as this, in our experience, is perceived by stakeholders to be a more achievable action and less complicated than amassing and extracting indicators and future data, which can be time consuming or may be unavailable. By changing the terminology of stage 5 of the process, it may make it seem less onerous and challenging and an easy step to add at the end of the HIA when closing it, particularly when a resource-light, rapid and smaller HIA has taken place.

### Challenges/Limitations of the Work

The HIA focussed on the major direct and indirect impacts only due to the scale and complicated nature of Brexit. It must be acknowledged however, that other impacts may not have been identified due to the lack of robust evidence available at that time. Indeed, new impacts came forth in the follow-up rapid review and update HIA report, such as NHS governance and waste management [38].

The HIA faced time constraints, as there was an agreed withdrawal date and deadline that needed information to inform planning, which was well before it happened and against a dynamic political landscape. This was exacerbated by the fact that the UK withdrawal from the EU was an unprecedented situation and the UK government’s negotiating position continued to remain uncertain. Thus, there was, and remains, little evidence on the exact implications of any scenario in relation to withdrawal. The withdrawal date also meant that the team had to carry out a comprehensive and robust assessment of a contentious and time-sensitive policy in a very short time; along with capacity and resourcing, timing and time constraints are noted as potential key challenges in carrying out HIAs [15,49]. The complexity of an HIA such as this highlighted the number of “hidden” additional hours involved in this dynamic HIA.

In addition, there was also no actual evidence of impact of Brexit because the UK had not yet withdrawn from the EU, and there was no previous work with respect to the public health implications, apart from a health technology assessment and health systems impact analysis [50]. Brexit had no comparable previous examples and there were no robust systematic reviews or experiences to draw upon to help inform decision-making and policy makers. Published evidence such as grey literature and opinion pieces are all highly contested and this was a challenge. As a result, editorials by single authors were excluded from the literature review. The evidence was weighted with peer-reviewed journals identified as part of the literature review. This was used as a platform on which to build the HIA appraisal, together with other identified strong evidence. Welsh population data and health statistics stood alongside this and were used to articulate whether a potential impact is relevant for Wales, and the likelihood of the size and susceptibility of the population in the absence of hard empirical evidence. For example, there are a number of major ports in Wales, making implications for that infrastructure from Brexit extremely likely. However, they could be of lower relevance and significance to other areas of the UK. Stakeholder evidence from interviews and the HIA workshop provided contextual knowledge only unless there was strong evidence derived from the literature to support it. For example, mental well-being was highlighted as a key determinant of health by the participants at the workshop and in interviews. There is evidence to support the mental well-being implications of pathways such as economic downturns [51], but the HIA did not actually identify any published qualitative research or health intelligence data analysis in relation to population mental health and well-being and Brexit.

Finally, constant political changes and shifts meant that the team had to react rapidly to these as they happened in real time and review the details to ascertain if the contents or context could change the contents of the HIA and the analysis constructed around the evidence. For example, Prime Minster Teresa May announced an agreement for withdrawal from the EU in early December 2018 [52]; the team had to review all the impact analyses to clarify if this may have changed any classification of impact or not.

## 5. Conclusions

This HIA demonstrates continued leadership by Wales in the field of impact assessment and “health in all policies”, and has demonstrated how HiAP can be practically implemented in our context. The work has been positively received. It created and solidified many high-level strategic advocates for HIA and HiAP across a wide range of national and local stakeholders. Forthcoming are HIAs responding to the COVID-19 pandemic and another on climate change in Wales.

It has transferable learnings and knowledge obtained from practice, which can be used by many nation states and devolved regions. Health policy leads, grappling with complex novel policies and needing a lens to think these through. It has demonstrated the “added value” of HIA to inform action in assessing policies, plans and projects at all levels, but particularly those in relation to dynamic and unknown significant events.

Finally, the Brexit HIA achieved its aim of identifying the potential positive and negative impact of the UK withdrawal and for whom, and provided a set of realistic, strategic and practical recommendations and actions (such as future research) that could be followed by decision makers and planners in an ever-changing political climate. Others could replicate this approach in a tangible way to do the same.

## Figures and Tables

**Table 1:** Terminology: Characterisation of impact.

• Positive—impacts that improve or maintain health status
• Negative—impacts that diminish health status
• Confirmed—actual direct evidence in existence
• Probable—more likely to happen than not, direct evidence but from limited sources
• Possible—may or may not happen
• Major—sufficiently great or important to be worthy of attention, noteworthy
• Moderate—average in intensity quality or degree
• Minimal—of a minimum amount, quantity or degree, negligible
• S = Short term—less than 1 year
• SM = Short to medium term—1–3 years
• ML = Medium to long term—3–5 or 10 years
• L = 10+ years

## Appendix A

**Table A1:** Example of summary screening paper—list of prioritised determinants of health to be investigated further.

Priority Determinants of Health	Potential Impact on Health	Potential Impact of Brexit	Comments/Rationale
**Health and Social Care**			
**Medicines and medical devices—regulation and licensing**	Yes	Negative	Medicines and Healthcare Regulatory Agency (MHRA) currently works with the European Medicines Agency (which is moving to Amsterdam)—post-Brexit position still unclear.
**Medical and professional qualifications**	Yes	Negative	United Kingdom Government (UKG) proposing to continue with existing arrangements but subject to agreement by European Union (EU).
**Staffing and recruitment**	Yes	Negative	British Medical Association (BMA) survey has shown that 45% of EU doctors considering leaving UK. Royal College of Nursing (RCN) and others have expressed concern about recruitment of nurses.
**Research and development**	Yes	Negative	UKG plans to discuss temporary mobility of scientists and researchers subject to agreement by EU. Access to EU networks may be reduced.
**Working hours and conditions**	Yes	Negative	UKG assurances on long-term continuation of existing workers’ rights under EU law questioned by RCN and others.
**Reciprocal healthcare**	Yes	Negative	UKG proposals for continuation of reciprocal healthcare for United Kingdom pensioners, the European Health Insurance Card (EHIC) scheme and cooperation on planned medical treatment subject to agreement by EU. Position on other types of reciprocal healthcare unclear.
**Fitness to practise**	Yes	Negative	UKG position on continuation of participation in EU Internal Market Information (IMI) alert system that records cases of professionals unfit to practise unclear.
**Health protection and security**	Yes	Negative	UKG proposals for ongoing work with key EU agencies on health security to enable information sharing and access to key datasets subject to agreement with EU.
**Rare diseases**	Yes	Negative	As above.
**Patient care and services**	Yes	Negative	Subject to staffing and recruitment and other issues.
**Pandemics and infectious diseases**	Yes	Negative	See Health protection and security.
**Clinical trials**	Yes	Negative	UKG proposals for continued participation in EU wide clinical trials subject to agreement with EU.
**Life sciences**	Yes	Negative	UKG proposals for continuation of existing or similar arrangements subject to agreement with EU.
**Medical students**	Yes	Negative and Positive	Potential vacancies created by fall in EU students may lead to increased opportunities for UK students.
**Health promotion**	Yes	Negative	UKG position unclear but Welsh National Health Service (NHS) Confederation calling for highest possible level of coordination on health promotion.
**Behaviours affecting health**			
**Food and diet**	Yes	Negative	UKG proposing common framework with EU but subject to agreement with EU. Implications of Trade Agreements, i.e., regulations and labelling.
**Alcohol, cigarettes, nonprescribed drugs**	Yes	Negative	Continuation of EU cross-border approach to antismoking measures through the Tobacco Products Directive (TPD) unclear. Implications of Trade Agreements, i.e., regulations and labelling.
**Mental well-being including:** **sense of control, resilience and participation**	Yes	Negative and Positive	Likely to dependent on population profile and attitudes/belief.Indirect impact of potential economic downturns post exit.
**Social and community influences**			
**Family organisations and roles**	Yes	Negative and Positive	Likely to depend on population profile and attitudes/belief. Impact of changes to immigration rules—impact on non-UK EU nationals who live and work in Wales.
**Citizen power and influence/social isolation/community networks/racism/hate crime**	Yes	Negative and Positive	Likely to depend on population profile and attitudes/belief.
**Living and Environmental factors**			
**Built environment**	Yes	Negative	A significant portion (>90%) of environmental legislation currently derives from EU law, giving rise to uncertainty in the short term.
**Housing**	Yes	Negative and Positive	Stock may increase if EU and other non-UK nationals leave but house prices may fall subject to market conditions.
**Noise/air quality/waste/community safety**	Yes	Negative and Positive	A significant portion of environmental legislation currently derives from EU law, giving rise to uncertainty in the short term, although the majority of environmental legislation is devolved.
**Economic Factors**			
**Workplace and working conditions**	Yes	Negative	The majority of health and safety legislation derives from EU law, giving rise to uncertainty.
**Economic inactivity/income/employment**	Yes	Uncertain	Dependent on market conditions/future trade agreements.
**Skills**	Yes	Negative and Positive	Expected short-term shortages may lead to increased opportunities for UK nationals to gain skills in longer term.
**EU Funding**	Yes	Negative	UKG has guaranteed funding for EU projects agreed before Brexit, but it is likely the majority of EU funding will disappear.
**Access and quality of services**			
**Careers advice**	Yes	Unclear	Will likely depend on economic conditions.
**Other caring services**	Yes	Negative and Positive	Short-term shortages may mean increased opportunities for employment for UK nationals in the longer term.
**Shops and commercial services**	Yes	Negative and Positive	Potential price rises and reduction in choice may led to closures, but opportunities may arise to create new businesses.
**Public amenities/services**	Yes	Negative	Will likely depend on economic conditions.
**Education and training**	Yes	Negative and Positive	Reduction in EU students may lead to increased opportunities for UK students.
**Information Technology**	Yes	Negative	UK proposals on a data protection agreement subject to agreement by the EU.
**Macro-economic, sustainability and governmental factors**			
**Gross Domestic Product**	Yes	Uncertain	May increase or decrease, depending on market conditions.
**Government policies**	Yes	Negative and Positive	Following transition from EU to UK law, policy impacts may be mixed.
**Economic Development**	Yes	Uncertain	May increase or decrease, depending on market conditions.
